# Some Popular Errors about Microbes

**Published:** 1902-07

**Authors:** Justin De Lisle

**Affiliations:** New York


### SOME POPULAR ERRORS ABOUT MICROBES.[1]

BY DR. JUSTIN DE LISLE, NEW YORK.

BACTERIOLOGY can be said to have had its beginning from the time the Dutch naturalist Leeuwenhoeck picked his teeth and examined the product of this research under what we would call to-day a small magnifying glass. This scientist was led far enough

[1] Read before The New York Institute of Stomatology, March 4, 1902.

by his investigations to detect bacteria in water, vegetable infusions, human saliva, the intestinal contents of flies, frogs, chickens, and man. In this latter observation he made the first step in modern human pathology, by noting the marked increase of bacteria in diarrhœa. These historical events took place about the year 1680, and it is certainly astonishing with what accuracy these microscopic organisms were described by the pioneer observers, descriptions that leave but little to add, even to-day with our highly perfected microscopes. Naturally I only refer to the few microbes that were observed and described, for bacteriology seems to have marched parallel with the improvements of the microscope. But even with all the advancement made in optics and mechanics, we have every reason to believe in the existence of microbes so small that our most powerful lenses do not reveal their presence, and beyond this there are no doubt other horizons not even explored in conjecture. Our microbic acquaintance now reaches to between six hundred and seven hundred different species, and yet out of the mass of facts collected and investigations that have been made in this department of botany no satisfactory system of classification has come out. Science to-day, in its relation to bacteriology, is in about the same position it was with chemistry in the latter part of the eighteenth century, and we wait upon an organizer like Lavoisier to collect and systematize this data and bring order out of chaos. Microbes are everywhere,—in the water, the air, upon our bodies, and even invading every cavity of the human economy. Environed by this myriad of apparently implacable enemies, it is a wonder that we are able to perform the simplest functions of life, we dare not go into the street, for there we are sure to meet some of our most terrible foes, like the bacilli of tetanus. In the carpets of our rooms, hanging to the walls of our apartments, and swarming over the furniture is this omnipresent host of destroyers. Even the middle of the ocean is infected, and from the highest point of Mount Blanc over three hundred different species of bacteria have been found. There seems to be no escape unless we would take to living in glass houses and stuff the chimneys with cotton, and even here we would not be safe from an affliction that is worse than an inoculation of the most virulent microbe. This disease is called *microbphobia.* It seems as though every advancement or innovation in any branch of science, by the alteration of our environment, has left its stigma upon us. We have the bicycle face and hump, the electric light and X-ray eye, the telephone ear, etc. Lastly, this terrible microbphobia, which may mean an imaginary fear of any or even all microbes. One of these poor afflicted imbeciles will not eat certain articles of food even after it has been heated many degrees above the life limit of any microbe; another will almost die from asphyxia in trying to keep from inhaling the expired air of an invalid, in spite of the fact that air expired from our lungs is absolutely sterile, and so on up to superstition and ridicule.

Inasmuch as microbes are everywhere, the three great divisions of nature are subject to attacks from bacteria and their products. The whole animal kingdom, from the most highly organized species, like that of man, to the most primitive amœba, and the vegetable kingdom from algæ to oak-trees, offer the richest nourishment and environment for the growth and multiplication of bacteria. The mineral kingdom is not ignored, and many examples can be cited where the life of a microbe depends upon mineral matter. According to these lines of restriction, we might come a little nearer to man's relation to bacteria, for we find that a certain class of microbes is pathogenic for animals alone; this number is forty-one. So out of nearly seven hundred different varieties of bacteria that infect the three kingdoms, only forty-one are able to attack the animal. Some of the microbes that have almost extinguished many species of animal, man not excluded, have no effect upon the vegetable world, and *vice versa*. The idea might be worth mentioning that it is quite probable many animals which have become extinct and have left only a history in fossil remains may owe their sudden extermination to a microbic invasion. But let us look again at these forty-one species of microbes that attack animals, and we find that only thirty-one are capable of infecting man. It is true there are yet many diseases that occasion great ravages in the human race, the microbe of which has not yet been isolated. However, for the time being let us be satisfied with these thirty-one species of microbes. Not a formidable number, it is true, but adversaries certainly worthy of all the acumen of science in calling forth every means of natural and artificial defence. Our ancestors in the middle ages went into battle covered from head to heel in a steel armor, but it would seem ridiculous for us to protect ourselves in any such manner, even if we substituted some lighter material. Nevertheless, nature has provided us with just such a protection, easy to carry and as impervious to microbes as the armor of Achilles was to the shafts of his opponents. The epithelial layer of the skin and mucous membrane constitutes our coat of mail. No pathologist, bacteriologist, or biologist has ever seen a microbe penetrating the unbroken epithelium. Covered as we are inside and out with this protective layer of cells, that are so closely adapted to one another that no microbe, no matter how small, can slip between them, and no matter how strong, can force them asunder, we are absolutely proof against the penetration of any microbe. Even more than this, if a microbe does, through any break in continuity, gain access into our interior, it is, in a great number of instances, immediately seized by the phagocytes and digested. What could be more ideal! Every tissue of the human body has its means, and effective means, too, of defence against all intruders, and all these self-protecting organs incased in an absolutely impenetrable membrane.

Viewed in this light the idea changes, and instead of asking how is it possible to escape from microbes, we wonder why we are ever infected. It is always in the unguarded and unexpected moment that invasion takes place, a scratch, a tarnished spot upon our armor, and the microbe enters. A lowering of vitality due to excesses or dissipation, and the phagocyte fails to seize or digest the intruder. In one of his moments of deepest depression Pasteur was greatly encouraged by the words of an eminent surgeon, who remarked that Pasteur's great work had taught surgeons to wash their hands. Yes, but not to skin them! It looks to-day as though asepsis had swung into an extreme.

Far be it from me to cry down reasonable asepsis or antisepsis, but I do sincerely wish to raise my voice against this extreme and even dangerous method of modern asepsis. To give the details of the toilet of a modern surgeon and a description of his boudoir in one of our modern hospitals would be beyond the scope of this address, but there are certain features of his preparation so striking as not to escape the notice of the most casual observer, like washing and briskly brushing with a stiff brush his hands and arms in strong soapsuds, chlorinated lime, sal soda, and then rinsing with alcohol, bichloride of mercury, etc. What does all this do but rub off his protective epithelium and lay him liable to infection.

The daily and professional papers have just chronicled the death of a distinguished surgeon in Baltimore, as the result of a slight wound from a bristle of his brush. But the modern surgeon is devoted to his profession and willing to sacrifice his well-being and the epithelium of his skin rather than lay his patient liable to any infection from dirty hands. Although, after all the details of this grand toilet have been strictly performed, he will confess that his hands are not really sterile; they are clean, yes; but not sterile, and the bacteriologist can prove the truth of this by making cultures from his well-washed skin. The technic of the operating-room is too complicated, and an error or oversight is almost sure to creep in. If the surgeon is painstaking and fastidious in his personal preparation, such, too, is the case with the patient. The seat of operation is scrubbed, soaped, and washed with the whole gamut of solutions, and lastly the skin for a large distance around the point of attack is scraped until, in some instances, the blood oozes through. Do you think this energetic procedure leaves a trace of epithelium, the natural wall of defence against infection? No; hardly a cell remains. There is nothing but a great unprotected surface, deprived of all external defence and gaping open in readiness to admit any and all microbes. But these surfaces are covered with antiseptic dressings.

A few days ago I witnessed a surgical operation in one of the most beautiful and modern hospitals in the world, here in the city of New York. Everything was aseptic. The operation finished, one of the nurses took out of an aseptic cupboard a roll of aseptic cotton, unwrapped its aseptic covering with her aseptic hands, but pulled out the aseptic pin that fastened this package with her septic teeth, teeth that were covered with microbes, the pus-forming microbes, perhaps other and more pathogenic microbes, and then applied this dressing to a freshly cut and largely denuded surface.

In view of just such trivial mistakes, that could lead to very serious consequences, many eminent surgeons are now considering the idea of abandoning extreme asepsis, substituting cleanliness and a preparatory vaccination of the patient with a bactericidal serum that will kill all microbes that might probably infect the wounds of their operations. If a man receives a gunshot wound or has a limb crushed in the street, it would certainly be good practice to give that patient an injection of antitetanic serum in anticipation of an infection from the bacillus of tetanus, for no matter how skilfully the operation in this case might have been performed, nor how carefully the antiseptic dressings be applied, tetanic infection would otherwise be possible.

Man at birth is free from bacteria; it is, however, only a few hours after he is born that his skin becomes peopled with a flora of microbes, but it is fully three days before any bacteria can be found in his intestinal cavity. Now, suppose this sterile condition which exists at the moment of his birth could be continued throughout the time of his life, would he be free from all diseases, at least from infectious diseases? This idea has suggested itself to experimenters, and the result of their observations is the only answer I can give. Two guinea-pigs were antiseptically taken from the uterus of the mother. One of the little pigs was nourished upon aseptic food, breathed aseptic air, and slept in an aseptic cage. It managed to live this aseptic life for thirteen days, but at the end of this period succumbed to marasmus and died. The autopsy of this animal proved the care with which this experiment had been performed by demonstrating the complete absence of bacteria. The other animal, surrounded by all the ordinary microbes that are contained in food, water, cages, and air, thrived and grew to maturity. Many series of like experiments have been performed upon guinea-pigs, and no animal has yet lived over sixteen days in an absolutely antiseptic condition. Experiments with tadpoles raised in sterile water, charged with sterile air, and fed upon sterile food has invariably ended in the death of the animal, whereas control tadpoles that have been kept in their normal surroundings have lived and developed. So, then, we are forced to the conclusion that bacteria have their uses, and some of them at least are really essential to the functions of life.

It has even been suggested, and the idea has been put into successful practice, that in stubborn cases of constipation, a disease, by the way, for the relief of which the materia medica offers no satisfactory remedy, to administer cultures of the coli communis, a microbe that normally inhabits the intestinal canal and has much to do with intestinal digestion and evacuation. The logic for this line of treatment is that constipation is a condition wherein the coli communis is very much diminished in number. In pursuing this course of treatment with cultures of coli communis it would perhaps be best to select a rather tame member of this group, for there are certain unmanageable varieties of coli that might excite the evacuation of more than one or two stools per day. This variability of properties is peculiar to many microbes, including some of the most harmless.

Inasmuch as we are called upon to admit the uses that bacteria are to the functions of the body, we can bring no greater argument to sustain this fact than the study of the bacteria of the mouth. Although the flora of the gastrointestinal tract is yet incompletely explored, much progress and many interesting facts have been brought to light. Thirty different species of bacteria have been isolated from the mouth. Of this number, nineteen, or almost two-thirds, are normal inhabitants, and, as we shall see in a moment, are essential to the functions of the buccal cavity. The remainder of this list may be classed as rare or accidental microbes that find their way into the mouth. This latter division can again be divided into harmless and pathogenic bacteria, so, when we at last come down to the really mischievous microbes of the mouth, their number is indeed small. Among the nineteen species with which we have to deal we find that five liquefy albumin, ten dissolve fibrin, nine digest gluten, seven ferment glucose, seven coagulate milk, ten digest caseine, nine change lactose into lactic acid, and seven infect cane-sugar, all processes of digestion and preparation of food for assimilation.

Still further than this, it is a well-known fact that wounds in the mouth heal with exceeding rapidity. It seems as though the contents of the mouth, secretions, microbes, or what not, stimulate the healing process. Witness, for example, how dogs lick their sores, cuts, and bruises, and how under this dressing they get well in a remarkably short period of time. I have just read of a passenger in a train who had the end of his finger mashed by shutting the door of his compartment as his train was leaving Genoa, Italy. This wounded man sucked his finger until he reached Geneva, in Switzerland, and the wound healed without further treatment. One of the most fertile and unexplored fields in bacteriology is that of bacterial association, or the influence that one microbe has over another.

Finally, we arrive at that branch of the medical profession which has to deal directly with the buccal cavity,—the dentist. Fortunately for him, his attention need not be turned towards the pathogenic microbes that may infect the mouth, for it is a well-known fact that normal saliva, though permitting the growth of harmless bacteria, is inhibitory for most all the disease-producing microbes, and as the former group is of use, his field of operation becomes very limited.

The mouth presents every facility for the multiplication of bacteria, such as heat, oxygen, moisture, nourishment, absence of light, and secure places to hide. But, upon the other hand, this cavity has a few disadvantages. First, the majority of substances that enter the mouth are sterile because they consist of well-cooked food, and, secondly, the natural defence against invasion and penetration of microbes as exercised by the mucous membrane that lines the mouth.

The mucous membrane of our body that is the most exposed to attacks from microbes is that of the conjunctiva. Our liability for infection of the conjunctiva dates from the second stage of labor to the moment we close our eyes forever. Added to warmth, moisture, oxygen, and nourishment, we have a constant planting of bacteria into the eye from the surrounding air, yet infection is comparatively rare. This immunity is not due to any bactericidal property of the tears, for this secretion exercises no harmful action upon bacteria, but to a purely mechanical process,—that of a constant washing of the mucous membrane of the eye with the lachrymal fluid. This conclusion has been obtained by introducing microbes directly upon the conjunctiva and observing after a short lapse of time their total disappearance from the eye into the nasal cavity. A continuation of this research has developed the interesting fact that a like mechanical defence is exercised in the mouth by the constant washing of that cavity with its own secretions.

The skin dislodges its pastes by a process of exfoliation which might be compared to a sort of dry washing. As to the fate of those microbes that succeed in penetrating through the superficial layers of the skin, but not far enough to become a prey to the ever-watchful phagocyte, their progress is barred by the underlying connective tissue, which immobilizes the invaders and checks their forward movement. Should a microbe be able to gain access into the connective tissue, it immediately provokes a thickening of the fibrinous elements, thus causing a localization of the microbian focus. In the skin this defence is amply illustrated by the slow progress of lupus as compared with tuberculosis of the lungs. Exfoliation and connective tissue resistance are both important factors in the defence of the mouth. The former, together with the washing of the mucous membrane by the secretions of the mouth, as explained above, serve as a constant and mechanical defence against certain microbes, for example, the bacillus of diphtheria, which is frequently found in the mouths of healthy persons. This same bacillus is also prevented from entering into the economy by connective tissue resistance, which by the production of fibrinous elements effectually seals up every port of entrance.

Many of the normal secretions of the body, following the particular diastase they contain, exert a powerful influence over microbes and their toxines. Among the lower order of animals this is illustrated by the intestinal canal of the crawfish and the common round worm that sometimes inhabits the digestive tract of man. Although the crawfish nourishes itself upon putrefying matter, and the round worm lives in and feeds upon a medium swarming with bacteria, yet the intestinal contents of these two animals is almost sterile. One gramme of pepsin from the human stomach will destroy fifty thousand fatal doses of the toxine of tetanus. Ptyaline from the saliva of man when mixed with a rapidly fatal dose of snake venom will render that poison perfectly harmless; *à propos* to this it is a common practice among certain African tribes to treat snake-bites by continually moistening the part with saliva.

But the most important property of the saliva is that of exciting positive chemiotaxis. By chemiotaxis we mean the attraction or repulsion that a microbe exercises towards the phagocytes. A phagocyte does not go directly to or from a microbe, nor does it take up and digest this same microbe without some underlying cause for this process. A certain microbe coming within the sphere of influence of the phagocytes immediately excites an attraction upon those cells and draws them on to its own destruction. Whereas another microbe, which to all outward appearances looks just as attractive and as good to eat, is ignored and scorned by the phagocyte, it is therefore passed by and allowed to continue its mischievous existence. In the former instance we say that the microbe exercised a positive or attractive chemiotaxis, but in the latter the process is reversed, a negative chemiotaxis is excited and the phagocyte repelled. There are several drugs that when added to microbes, or when injected into an animal some hours previous to an inoculation with bacteria, excite a negative chemiotaxis, and the microbe circulates in the system unmolested. Human saliva, however, happily acts as a powerful stimulant to positive chemiotaxis, and encourages the destruction of microbes. By the constant presence in the mouth of this excitant of positive chemiotaxis, a vast army of phagocytes is always in that region and the consequence is that the mouth is the best-guarded opening of our body. Therefore, should we undertake to wage a war against the normal bacteria of the mouth with a complicated technic of antiseptics, we would certainly do more harm than good.

Then why should the dentist wish to tear out his carpets and furniture, tile the floor, ceiling, and walls of his operating-rooms, bake his instruments until not a vestige of temper remains in them, in fact, imitate his *confrère,* the modern surgeon, and taking care not to omit many of his oversights? The logical response to this is, that all his surroundings and instruments being antiseptic, himself included, the patient has only to do with his own bacteria. There is no chance of a transfer of microbes from the operator to the subject nor from patient to patient. But all this is accomplished with ordinary cleanliness and is the daily routine practice of every dental operator in the country. The danger lurks in two places,—the omnipresent error that resides in a complicated technic and the over-confidence and false assurance in the antiseptic conditions of such surroundings that often lead to indiscretions, which, were it not for the protection that nature has provided, would end in serious results. This over-confidence which leads to carelessness is exactly the same as that which we place in our fire-proof hotels. So this antiseptic professional gentleman accomplishes no more than his carefully clean *confrère.* From the moment an instrument, whether from the autoclave or simple boiling water, touches the mouth of a patient they are upon an equal footing so far as the microbes of that particular individual are concerned. As to the transfer of the cause of a certain disease from the guilty to the innocent by operations in the mouth, grounds upon which many an honorable and careful member of this profession has been called upon to defend himself in the courts of law, it is in the light of recent investigation an utter impossibility, even when the most ordinary care has been observed. What if a microbe or two is imprisoned behind the filling of a tooth-cavity? it can do no harm, being surrounded as it is by a wall of dentine on one side and metal on the other; there, deprived of oxygen and proper nourishment, it must surely die. Then what use does this great parade of asepsis, apparatus, and antiseptic technic serve except as an advertisement or to encourage a microbphobic idea by perhaps a microbmaniac.

